# Empyema in spinal canal in thoracic region, abscesses in paravertebral space, spondylitis: in clinical course of zoonosis *Erysipelothrix rhusiopathiae*

**DOI:** 10.1007/s00586-012-2289-9

**Published:** 2012-04-17

**Authors:** Jarosław Andrychowski, Piotr Jasielski, Tomasz Netczuk, Zbigniew Czernicki

**Affiliations:** 1 Department of Neurosurgery, Mossakowski Medical Research Centre, Polish Academy of Sciences, Bielanski Hospital, Ceglowska Str 80, 01-809 Warsaw, Poland; 2 Medical University of Warsaw, Bielanski Hospital, Cegłowska Str 80, 01-809 Warsaw, Poland

**Keywords:** Zoonosis, *Erysipelothrix rhusiopathiae*, Abscesses in paravertebral space, Spondylitis

## Abstract

**Objectives:**

Erysipelas is an animal disease caused by Gram-positive bacteria *Erysipelothrix rhusiopathiae*. Among the domestic animals, domestic pig (*Sus scrofa f. domestica*) suffers most frequently from the disease in human environment. This is a typical animal-borne disease observed mainly in occupational groups employed in agriculture, farming (of animals and birds), fishing and manufacturing industry.

**Methods:**

We are presenting the clinical course of infection (*E. rhusiopathiae*) and discuss clinical forms. *E. rhusiopathiae* in humans may have the following clinical course: mild form of skin infection diagnosed as local erythema (erysipeloid), disseminated form of skin infection and the most serious form of infection of systemic course (endocarditis and sepsis). Mild skin infection and local erythema are the most common forms. Very rare case of animal-borne infection course has been presented in which after initial phase the disease was generalised to the abscesses formation in paravertebral space, spondylitis and empyema formation in spinal canal. In the presented clinical case, the patient was suffering from diabetes. It was probably an additional risk factor of the disease generalisation. Patient underwent drainage of empyema in spinal canal, after which his neurological status gradually improved. Antibiotic therapy was implemented and continued for 8 weeks. Such course of erysipelas was not previously described in the literature.

**Results:**

After therapy neurological status was improved. In follow MRI control exam empyema and spondylitis was successfully eliminated.

**Conclusions:**

Various complications of the disease, such as endocarditis and heart valves disturbances, are well known and are the most severe complications of the generalised infection. Proper targeted and long-term antibiotic therapy is crucial.

## Introduction

Erysipelas is an animal disease caused by Gram-positive bacteria *Erysipelothrix rhusiopathiae*. Among the domestic animals, domestic pig (*Sus scrofa f. domestica*) suffers most frequently from the disease in human environment. This is a typical animal-borne disease observed mainly in occupational groups employed in agriculture, farming (of animals and birds), fishing and manufacturing industry. Moreover, the people employed by animal waste segregation and utilisation companies belong to the risk group.

Erysipelas infection is a result of contact with infected animal, animal-borne contamination, animal-derived products or wastes.

Infection in humans may have the following clinical course: mild form of skin infection diagnosed as erythema (erysipeloid), disseminated form of skin infection and the most serious form of infection of systemic course (endocarditis and sepsis). Mild skin process and local erythema are the most common forms of infection. Despite sanitary control and advanced manufacturing industry, erysipeloid concerns specific occupational groups and is a serious economic problem in cases of *E. rhusiopathie* carrying by animals.

Usually observed and diagnosed dermatologic forms of disease are treated easily. More serious cardiological complications being a consequence of more serious forms of disease are rarely observed.

Very rare case of animal-borne (zoonosis) infection course in disseminated form of spinal canal empyema, perivertebral abscess and spondylitis was presented. In the presented case, no evidenced changes in heart muscle and circulatory system were confirmed clinically and by laboratory test results.

## Case report

62-year-old male patient working as a farmer and suffering from type 2 diabetes was admitted to the Neurology Department due to paraplegia of lower extremities, sphincters disturbances and sensation disturbances from the level of Th6. During the last 14 days preceding hospitalisation he moved with significant difficulty and reported weakness of the lower extremities. 4 days prior to admission he spent most of his time mainly in bed and suffered from paraplegia of lower extremities. After admission to the Neurology Department, initial diagnostics showed the presence of destructive changes within vertebral bodies, arches and spinous processes in the thoracic segment (Th5–Th6).

**Fig. 1:**
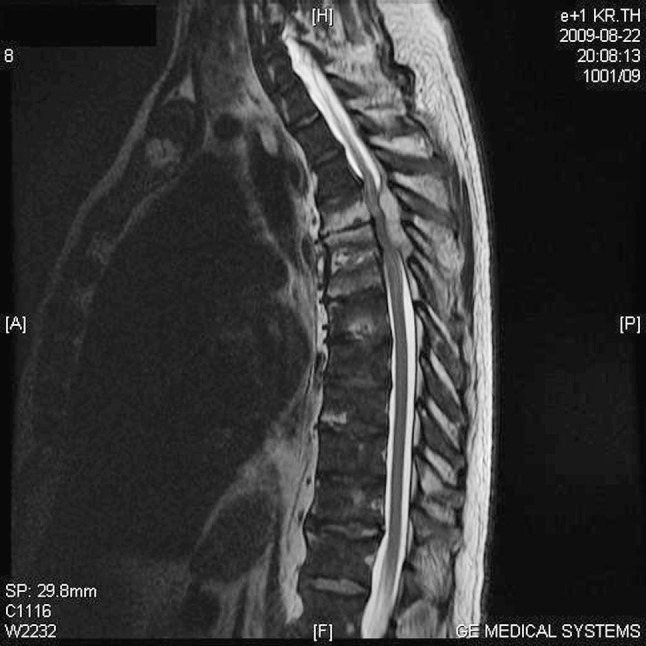
MRI examination before operation. Inflammatory process in the vertebrae and disc. Disc space narrowing and abscess in spinal canal

**Fig. 2:**
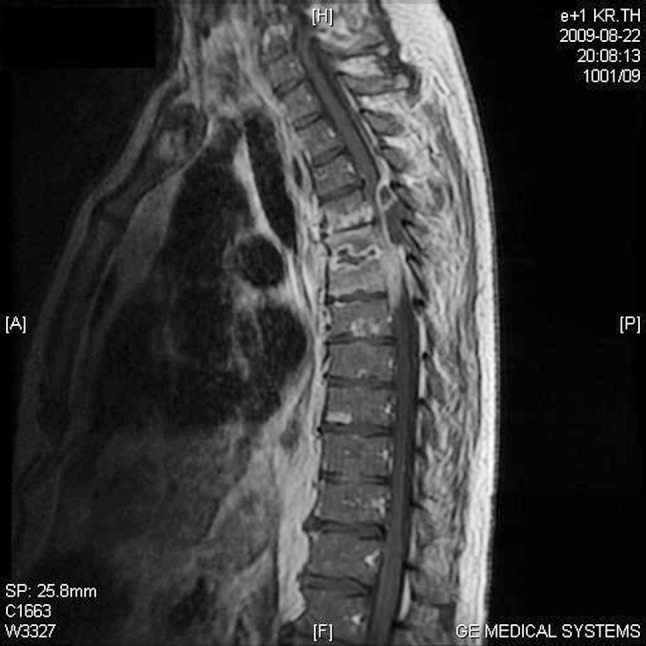
MRI examination before operation with contrast. Spinal cord compression

CT scans revealed destruction of the thoracic vertebras, infiltration changes in paravertebral soft tissues and pathological mass in the spinal canal. Initial diagnosis included infiltrative malignant tumour of the vertebral column. Consulting neurosurgeon recommended MRI examination. Examination revealed the presence of changes suggesting inflammatory process of the thoracic spine Th5–Th6, inflammatory changes within intervertebral discs and vertebral bodies and changes inside the spinal canal suggesting fluid mass empyema compressing the spinal cord (Figs. 1, 2).

Additional information collected from the patient and his family revealed that the patient suffered from the zoonosis, erysipeloid, 2 months priori to admission and was treated. Infection occurred after skin cut on the right leg with the fragment of the pig bone. Patient observed the presence of skin inflammatory process; subsequently, the process spread in the form of phlegmon of the right leg integuments. Patient was initially treated in the Department of Surgery and referred to the Orthopaedic Clinic due to skin phlegmon. Bacteriological tests revealed *E. rhusiopathiae*. Patient underwent drainage of the subcutaneous tissues. Targeted antibiotic therapy was implemented and continued for about 3 weeks after operation. As the family reported, patient reported back pain localised in the thoracic segment in the midline; nevertheless X-ray imaging of the spine was not performed, any local changes were observed within the paravertebral and superficial tissues. During admission and hospitalisation in the Neurosurgery Department, patient was suffered from glycaemia instability. As the normal levels of glycaemia were obtained, patient underwent operation. With regard to the process including distant vertebras and the risk of instability, double-level decompressing hemilaminectomy on the Th5–Th6 level was performed. Pus cistern of paravertebral localisation was found immediately after the incision of fascia lata. Infiltration of vertebral arches, vertebral joints and spinous processes was also observed. After flavectomy, epidural empyema was observed inside the spinal canal. Flow drainage of the operation field was performed for 5 days. In the post-operational period, cephalosporin was intravenously administered. Cultures and fragments of excised tissues were taken for histological examination. Local culture samples were collected from the wound during chronic infection as well as blood culture samples at the stage of systemic symptoms. *Erysipelothrix rhusiopathiae* were isolated. The patient was administered antibiotics based on culture outcomes and antibiogram. After spinal surgery, no pathological growth was observed in the collected samples—negative cultures.

On the 6th day after operation, control imagining (MRI) was performed which revealed evacuation of the empyema cavity.

**Fig. 3:**
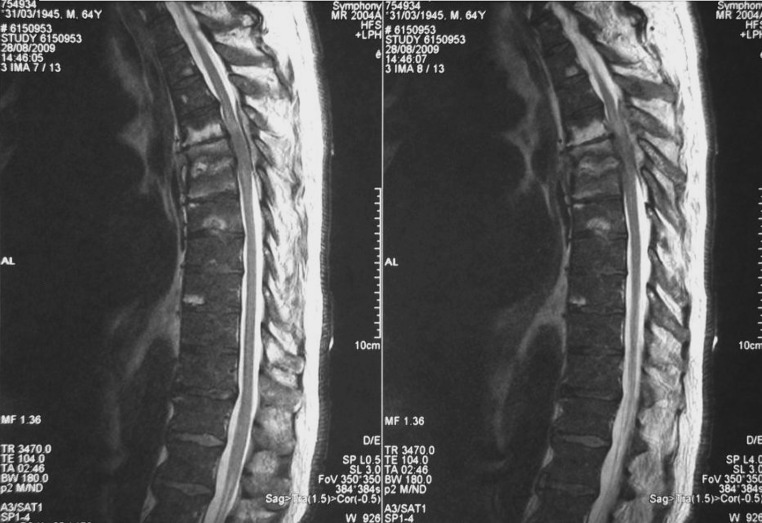
MRI postoperative control examination 6 days after operation. Decompression of spinal canal. Spondylodiscitis. Lesion of the spinal cord in postoperative level

Postoperative MRI examination revealed state after the hemilaminectomy and empyema drainage. The presence of inflammatory changes mainly in the Th5–Th6 segment in form of oedema of vertebral bodies, wedge-shaped vertebral bodies and uneven shape of intervertebral disc, spinal cord oedema, suspicion of inflammation, remnants of the empyema cavity in the spinal canal was observed. Clinically, unchanged neurological state was observed. Once the wound was healed, the patient was transferred to the Department of Infectious and Animal-borne Diseases and Rehabilitation with the brace (Fig. 3).

The clinical assessment after 16 months post operation revealed paraparesis of the lower extremities in neurological examination but the motor deficit was improved during this time. Patient was moving with a wheelchair.

**Fig. 4:**
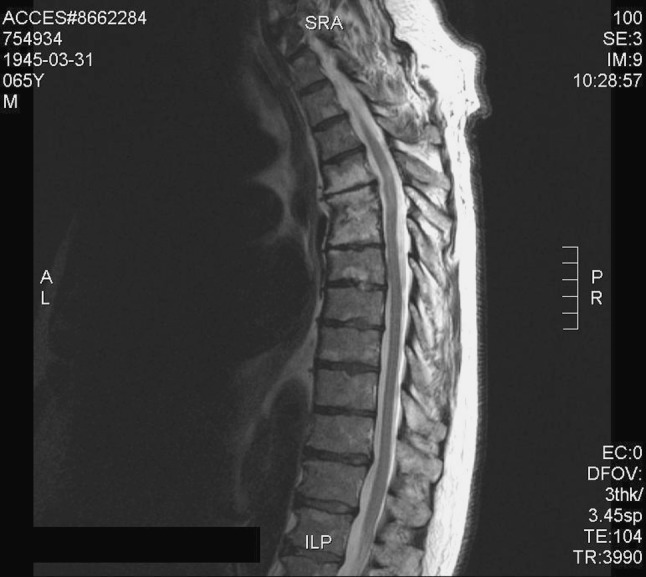
The follow-up MRI examination after 14 months post operation. Bone remodelling visible in Th5 and Th6 behind the damaged intervertebral disc as well as postinflammatory lesions within the Th4 and Th7 vertebrae. Spinal cord showing lesions at the level of Th4–Th5

**Fig. 5:**
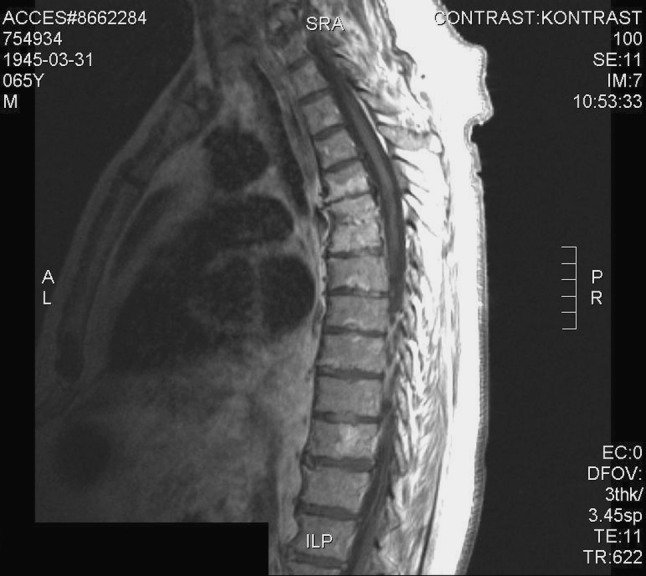
The follow-up MRI examination with contrast after 14 months post operation. Bone remodelling visible in Th5 and Th6 behind the damaged intervertebral disc as well as postinflammatory lesions within the Th4 and Th7 vertebrae. Spinal cord showing lesions at the level of Th4–Th5

A follow-up MRI scan was performed within 16 months postoperatively. There was visible; status post Th5–Th6 hemi laminectomy, status post spondylodiscitis involving Th4, Th5, Th6 and Th7 vertebrae. Bone remodelling visible in Th5 and Th6 behind the damaged intervertebral disc as well as postinflammatory lesions within the Th4 and Th7 vertebrae. Spinal cord showing lesions at the level of Th4–Th5 (Figs. 4, 5).

## Difficult zoonosis in the last time. Etiology and social problem

The definition of zoonosis was given in 1958 by WHO. Virchow was the first one to coin the term related to zoonosis as the problem in the human clinic in 1985, whereas as a veterinarian term it was introduced by W. Probstmayer in 1863.

Zoonoses are widespread all over the world with reference to the infection transmission ca. 200 types have been identified. The occurrence of zoonoses, exposition to the transmission factors refers to the population of both developing and developed countries and is a civilizational phenomenon. The prevention of possible infection paths of zoonotic infections is related to the civilizational level of a given country and its society. It is also related to an institution of veterinarian supervision. Lack of effective veterinarian supervision may result in the spread of zoonoses. Highly industrialised countries are equipped with a whole arsenal of services and resources allowing for effective prevention. However, it does not mean that zoonoses do not occur in the societies enjoying economic prosperity. They may occur in persons employed in agriculture, fishing and animal processing. They are related to groups of professional risk and persons disregarding supervisory procedures and educationally unaware. They may occur in pet breeders and, what is most interesting, they are also related to various culinary habits of various countries and cultures.

*Prions* In recent decades, diseases transmitted by prions have become a real challenge to medicine due to necessary mechanisms of prophylactics and little effectiveness of their treatment. Creutzfeldt–Jakob Disease (CJD) is a transmissible spongiform encephalopathy (TSE). Cases of bovine spongiform encephalopathy (BSE) have been also detected. CJD occurs in many etiological subtypes. Sporadic CJD–sCJD, genetic forms of human TSE (sTSE)—genetic forms of diseases like Gerstmann–Staussler–Scheinker–Syndrome, fatal familial insomnia. The iatrogenic form of these diseases iCJD is associated with the transmission of the infection among humans by implants or preparations containing hormone of human origin. The disease transmission during neurosurgical procedures is also possible, which has been documented in the literature. One of subtypes of spongiform encephalopathy in humans, subtype variant Creutzfeldt–Jakob Disease (vCJD), is evidently related to bovine spongiform encephalopathy (BSE) transmission onto humans [9, 13, 16, 20, 23, 33, 39, 43, 44].

The problem refers to developed countries. There are distinct suggestions that other types of spongiform encephalopathy may also be zoonosis, e.g. sCJD. In order to prevent the transmission of epidemiological factors in the recent decades, a monitoring system of animal breeding and epidemiological supervision has been developed in detail by certain internal regulations as well as on European Union level. It is also considered that presently recognisable cases of CJD in the world, due to the long incubation period of the diseases, may also be the consequence of the exposition to the pathogenic-zoonotic factor prior to the introduction of effective diagnostic methods of infections in animals.

*SARS* The another zoonosis known in recent years. Viral zoonoses are widespread, in recent years, SARS (severe acute respiratory syndrome) has been a large problem in Far East Asia. In the epidemiological aspect, the reason for SARS infection is coronaviridae, coming from a big Chinese cat so-called civet—a culinary delicacy from the region of Kanton. This disease afflicts the respiratory system, usually takes the form of a mild infection with a fever, however, severe cases of life-threatening pneumonia may occur [2, 7, 24, 30, 36].

*Avian influenza* (bird flu) recently, world news has been dominated by data related to the form of influenza of zoonotic origin. The epidemiology of the phenomenon has been related to the areas of Far East Asia, actions aimed at the counteraction of the epidemic spread consisting in the mass liquidation of chicken farms constituting a natural reservoir of the epidemic have represented a particularly drastic view. The infections of avian influenza are caused by A H5N1 virus, whose reservoir in nature is wild fowl, the virus is transmitted easily to poultry and shows very high pathogenicity. On poultry farms it undergoes numerous modifications and can be pathogenic to humans. The virus is widespread in the world, it is found in many organisms, also in humans. In certain situations, it becomes pathogenic. Poultry farms, particularly in Asian areas, are thought to be the source of modification of avian influenza virus gaining the properties of a pathogen that is dangerous to human organisms. In such situations, the monitoring of poultry farming as well as quick identification of an infection in humans is of extreme importance. Even slight number of diagnosed infections caused by avian influenza in humans, sometimes unfortunately of very severe course, constitutes particular information about the virus pathogenicity and consequently leads to the decision of liquidating a given reservoir [11, 21, 26, 29, 47].

*Swine influenza* (pig flu) has been initially so-called due to the type of virus isolated in swine organism and concurrently found in humans. Initially such name was pushed by the World Health Organisation. Falling ill is caused by the strain of virus A H1N1v/additionally marked with (v) to differentiate it from the types of influenza virus causing seasonal infections. This virus undergoes constant modifications. Infections in humans can be caused by the influenza virus that infects pigs and so far there have been such cases among persons having contact with animals, particularly among farm workers. However, recent infections found in the human clinic are mutated forms of viral infections where the virus contains elements of both human and avian influenza genome. In such situation, a pig farm can be the reservoir where a certain virus may undergo mutation and become dangerous to human and the infection course may be severe.

The above-mentioned viral diseases have been particularly noticeable in recent decades as diseases of civilization featured in mass media and also have constituted a high degree risk [1, 18, 32, 37, 38, 40].

In the human clinic also diseases of bacterial origin are present, to mention just the few most frequent ones: cat scratch disease, plague, anthrax, Lyme disease, brucellosis, tuberculosis, erysipeloid, salmonellosis, leptospiroses—Weil’s disease, malaria, Stuttgart disease and listeriosis. There are also parasitical diseases, of which the most frequent in developed countries is trichinosis, caused when humans are infected with by eating infected meat. The reasons of zoonoses include also protozoa and helminths.

## Discussion

Spinal infections are painful for patients and frustrating for doctors, it can be pyogenic (bacterial) granulomatous (tuberculosis or fungal) and after parasitic contamination. We divide pyogenic spinal infection from the clinical point of view to spondylodiscitis, septic discitis, vertebral osteomyelitis and epidural abscess. The most frequently isolated pathogen is *Staphylococcus aureus* [3, 6, 15, 25, 28].

Presented severe clinical course of zoonosis (erysipelas–*E. rhusiopathiae*) in form infection of vertebral column and spinal canal with accompanying severe neurological deficit is extremely rare. Probably the diabetes as additional risk factor caused generalisation of the skin infection and blood-derived abscess formation.

Human-acquired infection in majority of cases is limited to the inflammatory (skin process)—erythema (erysipeloid) of definite limitation. Diffused skin changes are rare, systematic infections are the rarest—endocarditis, which may cause permanent changes on the heart valves. Sometimes insignificant scratches, i.e. during the work, and erysipeloid, a disease of specific occupational groups, may in consequence lead to the disease generalisation as sepsis [4, 5, 8, 14, 19]. Cases of glomerulonephritis and meningitis in the course of *E. rhusiopathiae* infection are described [22, 27, 45]. Intra-articular infections in peri-operational period after endoscopy of the knee joint are described [42, 46].

Cardiological complications may result in infection—typical predilection to changes formation on the left heart structures, especially in aortic valve. Endocarditis in result of *E. rhusiopathiae* infection is burdened with high mortality about 33–38 % of cases compared to endocarditis of different aetiologies, mycotic aneurysms, valve damages, heart muscle empyemas are another complications of the infection. So far documented course with multiple paravertebral abscess formation, empyema formation in the spinal canal and inflammatory changes within the vertebral column was not fund in review of literature by the authors. The infection factor (*E. rhusiopathiae*) is a bacterium, gram-positive popular rod in land or water environment, occurring as pathogen, commensal or saprophyte in domestic and wild animals. A bacterium is also observed in fish and sea mammals. In animals, the infection may have character of severe sepsis, subacute course—the form of multifocal skin changes and chronic—with accompanying endocarditis and arthritis [5, 12, 17, 19, 31, 35]. Domestic pig is the most important reservoir of bacterium *E. rhusiopathiae* in the environment; the infecting agent is released from the infected animals by faeces, sweat and excreted mucous which pollute food and water. Aspect of carrying in about 20–40 % of healthy animal population, as the potential source of the infection transmission is stressed.

*Erysipelothrix rhusiopathiae* as the epidemiologic problem and exposition of occupational groups on the infectious factor are discussed in medical and veterinary literature. Occurrence of erysipelas infection became reduced due to safe technological processes in animals processing, specific safety procedures and, as a consequence, limitation of exposition of occupational groups on the infection [5, 8, 48, 49].

Human becomes infected by direct contact with animal-derived pollutions, infected animals, and via animal-derived products or remains of the animal bodies without veterinary supervision.

Generalised process with endocarditis formation is the most dangerous form of infection for the human being.

Clinical course in human depends most of all on proper diagnosis of initial phase of disease and implementation of targeted antibiotic therapy to prevent severe forms of infection. Despite effective therapy of skin manifestation of the disease, recurrences of disease and sepsis occurred. Bacterium is sensitive to majority of penicillins, cephalosporins and lincosamides. The resistance is presented towards aminoglycosides and vancomycin. Proper therapy of skin changes is of crucial importance—management in the initial phase of disease, so that the antibiotic therapy is proper and the insignificant scratch did not become an exit point of generalised infection [5, 10, 34, 41]. For severe forms of infection, sepsis and endocarditis, recommended antibiotic therapy should be administered by 4–6 weeks or longer depending on the form of the course and localisation of infection, i.e. in complicated purulent knee endoscopic surgery presented in the case report antibiotic therapy was administered for 16 weeks [19, 42, 45, 46].

Due to the way of transmission, environmental factors, potential bacteria carriers in presumed healthy animal population, erysipelas remains a disease of specific occupational groups that contact with animals and their tissues.

In animal-borne disease, the elimination of bacteria carriers is of crucial importance. It can be done with special safety procedures and local disinfection, as mentioned in publications, gave an effect in morbidity decrease. Sanitary supervision is important in morbidity reduction; however, the elimination of accidental injury of integuments is very difficult. Proper diagnosis and therapy prevent spreading of the infection.

In the presented case, two factors, which could be responsible for such course, occurred; diabetes which modifies the course of infection and probably too short period of antibiotic therapy only 3 weeks of the skin and subcutaneous process. Indications for long-term antibiotic therapy exist in case of complicated infections.

## Conclusions

1. Unusual, complicated courses caused by *E. rhusiopathiae* in patients with diabetes require prolonged antibiotic therapy.
2. Proper diagnosis and therapy of local skin changes are crucial.
3. Very rare clinical course of erysipelas in form of spondylitis, paravertebral abscesses and empyema in spinal canal was presented.
